# Supplementary material for: Learning curve and surgical outcome of robotic assisted colorectal surgery with ERAS program
Source: Sci Rep. 2022 Nov 29;12:20566. doi: 10.1038/s41598-022-24665-w (PMC9709162; doi:10.1038/s41598-022-24665-w)
Supplement: Supplementary file 2 — Supplementary Information 2. [file 41598_2022_24665_MOESM2_ESM.zip › RAL Raw data-Ñ[▒K/140/OP N_ñw╝╨░O▒Kñσ.pdf]

W83 / W83

臺中榮民總醫院
手術室護理記錄

[redacted]

基本資料

- 室別： 開刀房-17室，手術科別： CRS，手術類別： 常規，日期： 2022/01/07
- 進入時間： 0735 ； 麻醉開始時間： 0745 ； Time Out時間： 0910 ； 劃刀時間： 0911 ； 手術結束時間： 1250 送出時間： 1315 ；
- 麻醉方式： GE
  診斷：Malignant tumor of sigmoid colon
  手術名稱：Robot Assisted Colectomy(L)
  手術局部用藥：NIL
  術前預防性抗生素：
    - 抗生素名稱一： Veterin 1GM IVD/由麻醉護理師給予
    - 時間： 0855
    - 上次簽章: 何采穎
  ，追加預防性抗生素：
- 手術醫師： 蔣鋒帆，楊曙榮，陳威遠
- 麻醉醫師： VS 陳永銘
- 刷手護士A ： 黃宸盈，時間： 0735
  流動護士A ： 何采穎，時間： 0735

皮膚狀況

A-V Shunt： 無
術前： 完整
記錄者： 上次簽章: 何采穎
術後： 完整
記錄者： 何采穎

手術準備

手術臥式 單一手術臥式

-
    - 手術臥式： 截石臥式
    - 使用附件： 溫毯 40 ℃，腳架，其它： 海棉盒
    - 皮膚準備： 10%Aq Betadine，Hibiscrub，2% Chlorhexidine
    - 電燒： 使用電燒: 電極片位置： 其它： 迴路電極墊，記錄者： 上次簽章: 何采穎

止血帶T： 無

植入物

無

標本送檢

- 細菌培養 無
- 細胞學 無
- 冰凍切片 無
- 病理標本
  組織名稱一：rectum
  ,
  組織名稱二：distal cut end
  ,
  組織名稱三：IMA root
- 截肢 無

標本交付 標本： 3 袋，標本： 0 盒，標本： 0 瓶，細胞學： 0 管，Culture Tube： 0 管，被交付醫師： R 陳威遠，

- 記錄者： 上次簽章: 何采穎
- 時間： 1259

傷口術後敷料

傷口敷料： 傷口敷料： 無

- 請於圖上標示部位：

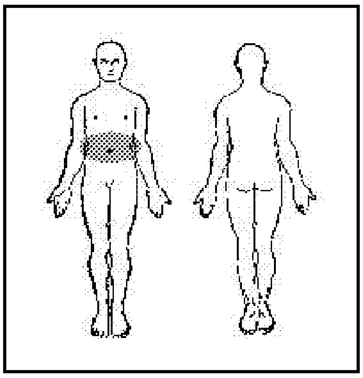

- 傷口敷料： MEPORE

導管

導管	天數	導管資訊	插入日期	插入時間	更換日期	拔除日期	拔除時間	拔除原因	預計拔除/更換	預計拔除/更換日期
Epidural PCA(1)	1	部位:脊髓;	2022/01/07	0745						
Sump NG Tube(1)	1	號碼:16 Fr.; 深度:55cm; 部位:胃;	2022/01/07	0810						
Double Lumen CVC(1)	1	號碼:8 Fr; 深度:14cm; 部位:jugular (右); 象限:RU;	2022/01/07	0820						
Foley(1)	1	號碼:16 Fr.; 部位:膀胱; 註記:ballon 10 ml;	2022/01/07	0835						
A-line (1)	1	號碼:20 Ga; 部位:左手;	2022/01/07	0815						

用物計數

- 手術中出血量： 少 c.c.
- 手術中尿量： 450 c.c.
-
  - 其它： 無

用物計數： 多切口同時縫合

- 1.器械： 正確 , 2.縫針： 正確 , 3.刀片 ： 正確 , 4.紗布： 正確 , 5.腹部墊： 未使用 , 6.紗球： 未使用 , 7.PEANUTS ： 未使用 , 8.紗條： 未使用 , 9.絲帶： 未使用 , 10.針頭： 未使用 , 11.Vessel loop： 未使用 , 12.Boots： 未使用 , 13.Jelco： 未使用 , 14.棉條： 未使用 ,
  - 第一次：
    - 刷手護士 ： 黃宸盈
    - 流動護士： 上次簽章: 何采穎
    - 時間： 1220

  ,
  - 第二次：
    - 刷手護士 ： 黃宸盈
    - 流動護士： 上次簽章: 何采穎
    - 時間： 1230

  ,
  - 第三次：
    - 刷手護士 ： 黃宸盈
    - 流動護士： 上次簽章: 何采穎
    - 時間： 1235

備註

備註： davinci 30度 XI scopy:No.2

- 記錄者： 上次簽章: 何采穎
- 時間： 0755

填寫護理人員 何采穎

正式摘要

W83 / W83

doctorRank:B
